# *Streptococcus pneumoniae* meningitis and the CNS barriers

**DOI:** 10.3389/fcimb.2022.1106596

**Published:** 2023-01-04

**Authors:** Eliza Gil, Emma Wall, Mahdad Noursadeghi, Jeremy S. Brown

**Affiliations:** ^1^ Division of Infection and Immunity, University College London, London, United Kingdom; ^2^ Francis Crick Institute, London, United Kingdom; ^3^ UCLH Biomedical Research Centre, London, United Kingdom; ^4^ Division of Medicine, University College London, London, United Kingdom

**Keywords:** *Streptococcus pneumoniae*, streptococcal infection, pneumococcal meningitis, meningitis, blood-brain barrier, blood-CSF barrier, neutrophil recruitment, pericytes

## Abstract

*Streptococcus pneumoniae* (SPN) is a globally significant cause of meningitis, the pathophysiology of which involves damage to the brain by both bacterial virulence factors and the host inflammatory response. In most cases of SPN meningitis bacteria translocate from the blood into the central nervous system (CNS). The principal site of SPN translocation into the CNS is not known, with possible portals of entry proposed to be the cerebral or meningeal blood vessels or the choroid plexus. All require SPN to bind to and translocate across the vascular endothelial barrier, and subsequently the basement membrane and perivascular structures, including an additional epithelial barrier in the case of the blood-CSF barrier. The presence of SPN in the CNS is highly inflammatory resulting in marked neutrophilic infiltration. The secretion of toxic inflammatory mediators by activated neutrophils within the CNS damages pathogen and host alike, including the non-replicative neurons which drives morbidity and mortality. As with the translocation of SPN, the recruitment of neutrophils into the CNS in SPN meningitis necessitates the translocation of neutrophils from the circulation across the vascular barrier, a process that is tightly regulated under basal conditions – a feature of the ‘immune specialization’ of the CNS. The brain barriers are therefore central to SPN meningitis, both through a failure to exclude bacteria and maintain CNS sterility, and subsequently through the active recruitment and/or failure to exclude circulating leukocytes. The interactions of SPN with these barriers, barrier inflammatory responses, along with their therapeutic implications, are explored in this review.

## 1 Introduction

*Streptococcus pneumoniae*, a Gram positive encapsulated bacterium, is part of the commensal upper respiratory tract flora and a globally significant cause of bacterial meningitis, accounting for the majority of cases in children and adults even in regions with high rates of vaccine coverage (Oordt-Speets et al., 2018). In spite of the existence of effective antibiotic treatment, pneumococcal meningitis continues to be associated with extremely poor outcomes: the mortality rate is around 20% even with optimal clinical management and approximately 50% of survivors are left with long-term sequalae, most commonly hearing loss (Hoogman et al., 2007; McGill et al., 2016). In most cases of bacterial meningitis, bacteria are thought to have translocated from the blood into the CNS during bacteraemia, although direct infection of the CNS through the cribriform plate or following trauma, surgery or extension of local infections of the head and neck also occur (Weber and Tuomanen, 2007; Hoffman and Weber, 2009). Once in the CNS, SPN triggers waves of apoptosis; initially due to direct damage by bacterial virulence factors, particularly secreted toxins, and subsequently to the exuberant host inflammatory response to the infection (Wache et al., 2015; Savva et al., 2016; Zierhut et al., 2017).

This potent inflammatory response is central to the pathogenesis of SPN meningitis. The CNS is highly intolerant of inflammatory responses which damage pathogen and host alike, particularly significantly the non-replicative neurons. For example, the high rate of sensorineural hearing loss after SPN meningitis is attributed to inflammatory damage to the cochlea (Du et al., 2006; Mook-Kanamori et al., 2011). The CNS is also contained within an anatomically closed compartment and is therefore vulnerable to oedema induced by the inflammatory response, which raises the intracerebral pressure leading to tissue ischaemia (Tuomanen et al., 1995; Nayak et al., 2012). Due to the vulnerability of the CNS to homeostatic disruption and inflammation, the cerebral environment is closely controlled with the movement of ions, molecules and cells tightly regulated by barriers at the interface of the brain with the blood and CSF (Abbott et al., 2006; Engelhardt and Coisne, 2011; Ampie and McGavern, 2022).

The blood–brain barrier (BBB) separates the blood from the cerebral interstitial fluid and brain parenchyma. The brain microvascular endothelial cells (BMECs) of the BBB have cell-cell junctional complexes, notably tight junctions, an absence of fenestrae, low levels of pinocytosis and low expression of leukocyte adhesion molecules, creating the ‘zona occludens’ within which there is marked immune specialization (Engelhardt and Sorokin, 2009; Daneman et al., 2010b; Daneman et al., 2010a). The cerebrovascular endothelium is closely associated with a layer of human brain vascular pericytes (HBVP) covering up to 70% of the abluminal endothelial surface, the densest of any vascular bed (Engelhardt and Sorokin, 2009; Armulik et al., 2010; Birbrair, 2018a). The basement membrane at this site is also structurally distinct, being composed of two layers separated by a perivascular space containing perivascular macrophages (Sorokin, 2010). External to the basement membrane are astrocyte endfeet, forming an additional barrier to entry of bacteria and leukocytes into the cerebral parenchyma from the circulation (Ampie and McGavern, 2022).

The blood-CSF barrier (BCSFB) separates the blood from the CSF at the choroid plexus in the ventricles (Solár et al., 2020). The vascular endothelial cells of the choroid plexus are fenestrated and lack tight junctions, enabling the egress of molecules and cells from the vessel into the surrounding subependymal space, which contains resident macrophages and infiltrating T cells. External to this are the epithelial cells of the ventricular cavity which possess tight junctions, limiting the movement of cells and molecules between the subependymal space and the CSF. (Ampie and McGavern, 2022). At its periphery, the brain is bounded by astrocytes forming the glia limitans superficialis, and external to this the meninges and further CSF spaces: the pia mater, the subarachnoid space, arachnoid mater, and then the dura mater. The endothelium of blood vessels on the cerebral surface and within the subarachnoid space is non-fenestrated with tight junctions, like the penetrating cerebral vessels, and the vessels are encircled by the pia mater, forming an additional barrier between the blood and CSF. The cells of the arachnoid mater express tight junction proteins, separating the CSF of the subarachnoid space and dura mater, which contains fenestrated blood vessels (Rua and McGavern, 2018).

These vascular barriers must be traversed by both pathogen and host leukocytes during SPN meningitis

## 2 *Streptococcus pneumoniae* invasion of the CNS

The principal site of translocation of SPN from the blood into the brain is not known and several sites have been proposed (Brown, 2015):

- From the post-capillary venules of penetrating cerebral vessels into the interstitial fluid in the perivascular space adjacent to the vessel, and thence the subarachnoid space, with which the perivascular spaces communicate, and the meninges.
- From the postcapillary venules of meningeal vessels in the subarachnoid space, into the subarachnoid CSF and hence the Virchow-Robins spaces and brain across the pia mater.
- From the blood vessels of the cerebral ventricles, across the choroid plexus epithelium into the CSF and hence to the brain and meninges.

The relative contributions of each of these is not known and remains controversial (Rodriguez et al., 1991; Zwijnenburg et al., 2001; Iovino et al., 2013). There are likely to be spatiotemporal differences in the sites of bacterial translocation during the course of the disease: in mouse models of bacteraemia-derived meningitis, SPN initially adheres to the subarachnoid vessels, and subsequently to increasingly internal cortical areas, but were not the choroid plexus until considerably later in the infection (Iovino et al., 2013).

Pathogens can breach the vascular endothelium *via* transcytosis through endothelial cells, paracytosis between endothelial cells, or inside infected leukocytes recruited into the CNS through a ‘Trojan horse’ mechanism (Doran et al., 2013; Anil and Banerjee, 2020). The interaction of SPN with the vascular endothelium initially requires molecular interactions between the pathogen and endothelial cell surface receptors. These interactions and the subsequent bacterial translocation can be augmented by activation of BMECs by inflammatory mediators, which occurs early in response to SPN in the bloodstream (Ring et al., 1998; Iovino et al., 2013). SPN neuraminidase A induces an inflammatory response in BMECs, facilitating bacterial adhesion and subsequent translocation across the endothelium (Banerjee et al., 2010). SPN adhesion to the cerebrovascular endothelium requires binding of the SPN surface protein PspC (also called CbpA) to endothelial laminin receptors (Orihuela et al., 2009). PspC also binds the human polymeric immunoglobulin receptor, which additionally binds the pneumococcal pilus 1-adhesin RrgA, strengthening adhesion of SPN to the endothelial surface (Iovino et al., 2017). SPN surface enolase, a glycolytic enzyme, also binds endothelial surface-bound plasminogen, further enhancing adhesion to endothelium (Bergmann et al., 2013). Binding of SPN phosphorylcholine (PCho) to endothelial platelet activating factor receptor then mediates vacuolar uptake of the bacteria (Cundell et al., 1995; Cundell et al., 1996; Radin et al., 2005; Fillon et al., 2006).

During transcellular passage, SPN is endocytosed in a clathrin or caveolae dependent method, or by a novel dynamin-independent mechanism (Surve et al., 2020). The majority of bacteria are then killed within phagolysosomes, but some survive and are transferred *via* β-arrestin mediated cytoskeletal changes either back to the endothelial luminal surface or across the cell to the abluminal surface, with subsequent exocytosis of the bacteria into the perivascular space (Ring et al., 1998; Gradstedt et al., 2013). The survival of SPN appears to be influenced by both the level of pneumolysin expression, with high levels of toxin production disrupting the phagosome, as well as the endocytosis mechanism, with dynamin-independent endocytosis conferring a survival advantage by avoiding lysosomal degradation (Surve et al., 2018; Surve et al., 2020). The SPN capsule, the main virulence factor, is thought to impair SPN adhesion to host cells, but recent data suggest it may actually enhance SPN transcytosis and tissue invasion (Brissac et al., 2021).

In addition, SPN may translocate paracellularly. SPN is able to bind CD31 (PECAM-1) at the endothelial cell-cell junctions, which could enable para-cellular translocation across the BBB (Iovino et al., 2014a; Iovino et al., 2014b). Furthermore, the SPN toxins, pneumolysin, a pore forming toxin, and α-glycerophosphate oxidase, which drives the production of H_2_O_2_, cause structural damage to, and induce apoptosis in, endothelial cells, undermining the structural integrity of the vascular endothelial barrier which may facilitate further entry of SPN into the CNS (Quagliarello et al., 1986; Zysk et al., 2001; Wellmer et al., 2002; Hirst et al., 2008; Mahdi et al., 2012; Zhou et al., 2012; Shaji et al., 2019).

Once across the cerebral vessel endothelium, SPN must traverse the basement membrane (BM) and other perivascular structures, a process that remains poorly described. SPN uses surface receptors to bind host plasminogen, a protease able to cleave BM components (Eberhard et al., 1999) and plasminogen activation products are found in the CSF during bacterial meningitis and correlate strongly with BBB permeability (Winkler et al., 2002). SPN also expresses hyaluronan lyase (Jedrzejas, 2007), which degrades constituents of the extracellular matrix (ECM) and may therefore facilitate traversal of the BM (Zwijnenburg et al., 2001). Clinical SPN meningitis isolates show higher levels of expression of hyaluronan lyase than carriage isolates, indicating the importance of this enzyme in mediating invasiveness (Kostyukova et al., 1995). In addition to basement membrane, to penetrate the cerebral vasculature SPN must also cross the pericyte layer, perivascular space, and breach the encircling astrocyte endfeet to access the cerebral parenchyma. Pneumolysin induces astrocytic cell shape changes due to cytoskeletal reorganization, which can cause endfoot retraction (Förtsch et al., 2011; Hupp et al., 2012). However, the interaction of bacterial meningitis pathogens with the HBVP remains poorly understood.

The blood-CSF barrier at the choroid plexus is also hypothesized to be a possible site of SPN infiltration of the CNS, and another meningitis-causing streptococcus, *Streptococcus suis*, can indeed translocate across the choroid plexus epithelium and disrupt the BCSFB (Tenenbaum et al., 2006; Tenenbaum et al., 2009; Hoffman and Weber, 2009). However, as already discussed, in mouse models of bacteraemia-derived meningitis SPN could only be identified at this site late in the infective process (Iovino et al., 2013). The BCSFB is structurally distinct from the BBB: the vascular endothelium here lacks tight junctions and is fenestrated so may pose less of a mechanical challenge to bacterial translocation. However, extravasating bacteria enter the subependymal space which contains resident macrophages and patrolling T cells and subsequently have to cross the epithelial layer, where the cells are joined by tight junctions, in order to access the CSF (Ampie and McGavern, 2022).

## 3 Leukocyte recruitment across brain barriers during SPN meningitis

The presence of SPN in the CNS has been described to generate ‘some of the most powerful inflammatory responses known in medicine’ (Weber and Tuomanen, 2007). This potent inflammatory response, principally mediated by the influx of neutrophils from the blood into the CNS, is sufficient to induce the full clinical meningitis syndrome in animal models (Tuomanen et al., 1985). Neutrophils, therefore, while central to the defence against bacterial infection, also play a key role in pathogenesis and represent a therapeutic target in SPN meningitis.

Several aspects of neutrophil recruitment in SPN meningitis remain poorly described, limiting our ability to target this process therapeutically. As with the translocation of SPN from the blood into the CNS, the recruitment of neutrophils from the circulation into the CNS necessitates translocation across the BBB and/or BCSFB, the relative contributions of which remain unclear (Engelhardt et al., 2001; Ransohoff et al., 2003; Mrass and Weninger, 2006). In addition, which cells detect SPN and generate the subsequent inflammatory cascade to stimulate neutrophil recruitment across the vascular endothelium into the CNS is not yet well elucidated.

The endothelial layer represents the first tissue barrier to the extravasating neutrophil and the transendothelial migration of neutrophils is regulated by both the endothelial cells as well as the perivascular cells and structures (Weninger et al., 2014; Kim and Luster, 2015). Neutrophil translocation across vascular endothelial layers occurs *via* a well described multi-step process termed the ‘leukocyte adhesion cascade’ initiated through interactions with the vascular endothelium (Ley et al., 2007). How this process is initiated in SPN meningitis has not yet been described: the specific site of neutrophil transmigration in SPN meningitis, across the BBB or BSCFB, is not clear, nor are the mechanics by which neutrophils traverse these barriers, which is likely to be influenced by barrier integrity. The BMECS of the BBB imposes specific challenges to the extravasating leukocyte. BMECs constitutively express only low levels of leukocyte adhesion molecules as well as forming tight junctions at cell-cell interfaces (Daneman et al., 2010b; Daneman et al., 2010a). Elsewhere in the vasculature, the majority of leukocytes extravasate paracellularly, however, in mouse models of neuroinflammation the majority of T cells extravasate the cerebral vasculature transcellularly, likely due to the tight junctions between BMECs (Engelhardt and Ransohoff, 2012). The vascular endothelium of the BCSFB does not have tight junctions, however, the epithelial cells of the choroid plexus do, and pose a considerable mechanical barrier to the passage of leukocytes (Ampie and McGavern, 2022). In mouse models of cerebral ischaemia and traumatic brain injury neutrophils have been shown to be able to extravasate at the choroid plexus, however the BBB of the leptomeninges and penetrating vessels was the predominant site of neutrophil extravasation in ischaemia (Szmydynger-Chodobska et al., 2009; Otxoa-de-Amezaga et al., 2019).

Once across the BBB BMEC layer, neutrophils must traverse the pericyte layer, the two layers of the BM and the perivascular space (Sorokin, 2010). In mouse models of sterile muscle or skin inflammation, pericytes have a role in regulation of neutrophil extravasation *via* the secretion of chemokines and pro-inflammatory cytokines (Proebstl et al., 2012; Stark et al., 2013; Kim and Luster, 2015; Girbl et al., 2018). Pericytes secrete a broad repertoire of molecules including leukocyte adhesion molecules and inflammatory cytokines, including chemokines, notably the potent neutrophil chemokine CXCL8 (Alcendor et al., 2012; Guijarro-Muñoz et al., 2014; Birbrair, 2018b). HBVP-BMEC interactions are important for cerebral vascular barrier function, and HBVP also have both pro and anti-inflammatory interactions with BMECs (Engelhardt and Sorokin, 2009; Armulik et al., 2010; Pieper et al., 2014; Banks et al., 2018; Birbrair, 2018a). Perivascular macrophages (PVMs) in the perivascular space can promote neutrophil recruitment during infection in other tissues (Abtin et al., 2014; Schiwon et al., 2014; Kim and Luster, 2015). In animal models of SPN meningitis, the depletion of tissue-resident macrophages results in decreased CSF leukocytosis, while astrocytes and microglia can be activated by SPN supporting their potential roles in neutrophil recruitment across the BBB (Polfliet et al., 2001; Weber and Tuomanen, 2007; Iovino et al., 2013). We have demonstrated HBVP to be highly responsive to macrophage signalling downstream of SPN stimulation, driving the secretion of inflammatory mediators, including the neutrophil chemokine CXCL8, which is translocated across the endothelial barrier and drives neutrophil translocation across the endothelial barrier (Gil et al., 2022). Together, these data provide support for the role of perivascular cells in the initiation and amplification of the CNS inflammatory response during SPN meningitis.

## 4 Therapeutic implications

Corticosteroids are already used as a non-specific adjuvant anti-inflammatory therapy alongside antibiotics (Brouwer et al., 2010). Challenges remain in this approach: the lack of a comprehensive understanding of the myriad inflammatory signalling pathways involved in the inflammatory response to SPN meningitis, as well as likely signalling redundancy, has hindered the ability to specifically target neutrophil recruitment in SPN meningitis. The is exemplified by the fact that in animal models of SPN meningitis, intrathecal injection of TNF is sufficient to induce CNS neutrophil recruitment, however, the concomitant administration of anti-TNF antibodies alongside SPN only diminishes neutrophil recruitment and does not abrogate it (Saukkonen et al., 1990). Consistent with this, animal models of SPN meningitis support a role for IL-1β, IL-6 and complement in the recruitment of neutrophils in SPN meningitis (Saukkonen et al., 1990; Koedel et al., 2002; Paul et al., 2003; Griffin et al., 2007). BMECs, and HBVP, are sensitive to many of the inflammatory mediators secreted by canonical innate immune cells in response to stimulation with SPN, with HBVP producing a broad repertoire of chemokines in response stimulating neutrophil recruitment across the BMEC barrier (Gil et al., 2022). It therefore appears that therapeutic interventions targeting neutrophil recruitment in SPN meningitis would need to act at the point at which these myriad pathways converge in order to be successful, if such a point exists.

The presence of chemokines bound to the vascular endothelial surface is key to the leukocyte adhesion cascade (Nourshargh and Alon, 2014). Many chemokines have been detected in the CSF of patients with *S. pneumoniae* meningitis including CCL2, CCL3, CCL4 CCL8, CCL15, CCL18, CCL20, CXCL1, CXCL5, CXCL7, CXCL8 and MIF (Spanaus et al., 1997; Zwijnenburg et al., 2003; Østergaard et al., 2004; Kastenbauer et al., 2005; Holub et al., 2007; Coutinho et al., 2013). Hence, a broad repertoire of neutrophil chemokines is secreted in SPN meningitis, which, along with inflammatory cytokines and bacterial products, interact to modulate neutrophil responses in the CNS (Wall et al., 2020). The BBB allows only minimal movement of molecules across its surface, and chemokines are not able to move passively across the endothelial barrier but must be actively transported from the abluminal to luminal surface if they are recruit leukocytes from the circulation. This process appears to be principally mediated by atypical chemokine receptor 1 (ACKR1), which is expressed on the cerebrovascular endothelium (Middleton et al., 1997; Hub and Rot, 1998; Pruenster et al., 2009; Minten et al., 2014). ACKR1 was first identified as an erythrocyte blood group antigen, Duffy antigen, and is alternatively known as Duffy antigen receptor for chemokines (DARC), or CD234 (Cutbush et al., 1950). The ACKR family exhibit highly promiscuous ligand binding, preferentially binding most inflammatory but not homeostatic chemokines (Gardner et al., 2004). Upon ligand binding, BMEC ACKR1 internalizes and transcytoses bound chemokines from the abluminal to luminal apical surface, where they stimulate neutrophil transmigration (Pruenster et al., 2009). ACKR1 may therefore represent a candidate therapeutic target in reducing chemokine expression on the vascular endothelial surface, however, the mechanism by which it works has not been fully elucidated, and in addition blockade of ACKR1-bound chemokines appears to enhance reverse neutrophil transmigration, with pro-inflammatory neutrophils returning to the circulation (Luo et al., 1997; Middleton et al., 1997; Zhao et al., 2011; Girbl et al., 2018).

## 5 Conclusion

The barriers of the brain: the BBB between the blood and extracellular fluid of the brain parenchyma, and the BCSFB between the blood and CSF in the ventricles, are central to the pathogenesis of SPN meningitis both through the failure to exclude the pathogen and subsequently as the sites by which circulating neutrophils transmigrate into the CNS, where they cause catastrophic damage. Neutrophil recruitment, and the upstream inflammatory cascade that drives it, represents a novel therapeutic opportunity. Unfortunately, there appear to be many upstream mediators of this process as well as marked signalling redundancy, limiting the ability to target this stage of the inflammatory cascade. The cerebrovascular endothelium is the key interface between tissue and circulating leukocytes, and the chemokines binding to the endothelium seems to be essential for the leukocyte adhesion cascade. Chemokines produced from resident immune cells and pericytes in the CNS in response to SPN are translocated across the cerebrovascular endothelium by ACKR1, which represents a point of convergence of several inflammatory pathways.

## Author contributions

EG, EW, MN and JB conceived the manuscript. EG and JB wrote the text and figures, which were critically reviewed by EW and MN. All authors contributed to the article and approved the submitted version.
